# Lubeluzole Repositioning as Chemosensitizing Agent on Multidrug-Resistant Human Ovarian A2780/DX3 Cancer Cells

**DOI:** 10.3390/molecules27227870

**Published:** 2022-11-15

**Authors:** Maurizio Viale, Giovanni Lentini, Rosaria Gangemi, Patrizio Castagnola, Gualtiero Milani, Silvia Ravera, Nadia Bertola, Antonio Carrieri, Maria Maddalena Cavalluzzi

**Affiliations:** 1 UOC Bioterapie, IRCCS Ospedale Policlinico San Martino, Largo R. Benzi 10, 16132 Genova, Italy; 2 Dipartimento di Farmacia-Scienze del Farmaco, Università degli Studi di Bari ‘Aldo Moro’, Via E. Orabona 4, 70126 Bari, Italy; 3 Dipartimento di Medicina Sperimentale, Scuola di Scienze Mediche e Farmaceutiche, Università di Genova, Via De Toni 14, 16132 Genova, Italy

**Keywords:** antiproliferative activity, apoptosis, doxorubicin, drug repositioning, lubeluzole, synergism

## Abstract

In a previous paper, we demonstrated the synergistic action of the anti-ischemic lubeluzole (Lube S) on the cytotoxic activity of doxorubicin (Dox) and paclitaxel in human ovarian cancer A2780 and lung cancer A549 cells. In the present paper, we extended in vitro the study to the multi-drug-resistant A2780/DX3 cell line to verify the hypothesis that the Dox and Lube S drug association may potentiate the antitumor activity of this anticancer compound also in the context of drug resistance. We also evaluated some possible mechanisms underlying this activity. We analyzed the antiproliferative activity in different cancer cell lines. Furthermore, apoptosis, Dox accumulation, MDR1 downregulation, ROS, and NO production in A2780/DX3 cells were also evaluated. Our results confirm that Lube S improves Dox antiproliferative and apoptotic activities through different mechanisms of action, all of which may contribute to the final antitumor effect. Moderate stereoselectivity was found, with Lube S significantly more effective than its enantiomer (Lube R) and the corresponding racemate (Lube S/R). Docking simulation studies on the ABCB1 Cryo-EM structure supported the hypothesis that Lube S forms a stable MDR1-Dox-Lube S complex, which hampers the protein transmembrane domain flipping and blocks the efflux of Dox from resistant A2780/DX3 cells. In conclusion, our in vitro studies reinforce our previous hypothesis for repositioning the anti-ischemic Lube S as a potentiating agent in anticancer chemotherapy.

## 1. Introduction

Lubeluzole (Lube S, Figure S1) is an enantiopure benzothiazole derivative that demonstrated neuroprotective activity in preclinical models of ischemic stroke, although several clinical trials showed scarce beneficial effects in humans. Its activity has been associated with different mechanisms of action, including inhibition of glutamate-activated nitric oxide (NO) synthesis, inhibition of glutamate release, blockade of voltage-gated calcium channels, and allegedly blockade of voltage-gated sodium channels (VGSCs) [1,2,3,4,5]. The anti-ischemic activity of Lube S was also related to possible epigenetic inhibition of NO synthase (NOS) and calmodulin (CaM) activities [2,6].

Recently, we developed an affinity capillary electrophoresis (ACE) method to assess the apparent dissociation constants between CaM and nonpeptidic ligands [6]. This method showed Lube S affinity for CaM, which correlates with its inhibitory activity on Ca^2+^/calmodulin-dependent kinase II (CaMKII). Docking simulations also investigated the possible binding modes of Lube S to CaM.

We also established that Lube S enhances the activity of some anticancer drugs in sensitive cells.

We demonstrated that Lube S synergizes with doxorubicin (Dox) and paclitaxel on human ovarian adenocarcinoma A2780 and lung carcinoma A549 cells [7]. This activity might stem from some of the mechanisms described above, particularly the inhibition of calmodulin activities [6] and the voltage-gated sodium channel (VGSC) blocking activity. This synergistic effect was present in a wide range of concentrations, with the lowest limit more than 100 times lower than IC_50_ values for VGSC [8,9] and at least 40 times lower than human plasma concentrations relevant for both anti-ischemic activity and cardiotoxic concern [10]. Interestingly, this last result indicated Lube S as a potentiating compound of Dox or paclitaxel antitumor activity with low toxic liability.

The effectiveness of chemotherapy strategies is still low for most cancer types, often because of cancer cell drug resistance. The acquired resistance that follows drug treatment is characterized by different mechanisms [11,12,13,14]. One of these mechanisms, called multidrug resistance (MDR) [14,15], confers acquired cross-resistance to a family of functionally and structurally unrelated chemotherapeutics. MDR1, characterized by an overexpression of the ABCB1 or Pgp-170 drug transporter, has been extensively studied to find compounds able to counteract its action using cell lines often selected by exposure to increasing concentrations of cytotoxic agents [16].

The problem of cell resistance and the wide range of mechanisms of action for Lube S prompted us to study the possible enhancement of Dox activity in multidrug-resistant A2780/DX3 cells, which overexpress the ABCB1 drug transporter [17]. In this context, we also studied the presence of possible concomitant mechanisms contributing to Lube S chemosensitizing activity.

## 2. Results

### 2.1. Inhibition of Cell Proliferation by Lube S, Lube R, and Lube S/R

The treatment of different sensitive cell lines with Lube S, Lube R, and Lube S/R (Table 1) showed good antiproliferative activity, mainly ranging from 4.7 ± 1.5 µM to 29.6 ± 4.0 µM, as 30 μM was considered the arbitrary limit to define a pharmacologically significant activity. Only Lube R gave in A549 cells an IC_50_ higher than 30 µM.

The A2780/DX3 cells were always less sensitive to all Lube forms than to sensitive A2780 cells (Table 1). Resistance became pharmacologically relevant with Lube S/R, as RI was 3.4 (>2.5 is the arbitrary limit to define drug resistance). Nevertheless, for all the studied compounds, the IC_50_ values were consistently higher than the mean IC_50_s calculated for Dox for both A2780 and A2780/DX3 cells (RI = 80) or the other cell lines. Furthermore, Lube S was more active than Lube R or their racemic mixture in most cell lines (Table 1).

As regards normal noncancer cells, activated PBL (*n* = 3) showed a low sensitivity to Lube S, their IC_50_s ranging from 32.7 ± 6.9 µM to 49.5 ± 2.5 µM (mean ± SD: 40.8 ± 6.9 µM). Excluding A549 cells, where Lube S did show a nearly absent antiproliferative activity (IC_50_: 28.8 ± 10.0 µM), on average, our compound was on PBL cells about six to seven times less active than on the tumor cell lines used as target (Table 1).

Based on the same experiments, we also calculated the concentrations of studied molecules giving 5%, 10%, 30%, 50%, and 75% cell proliferation inhibition in A2780/DX3 cells, used for some of the following experiments (Table 2).

### 2.2. Inhibition of A2780/DX3 Cell Proliferation by Dox Associated with Lube Enantiomers or the Racemate

**Figure 1:**
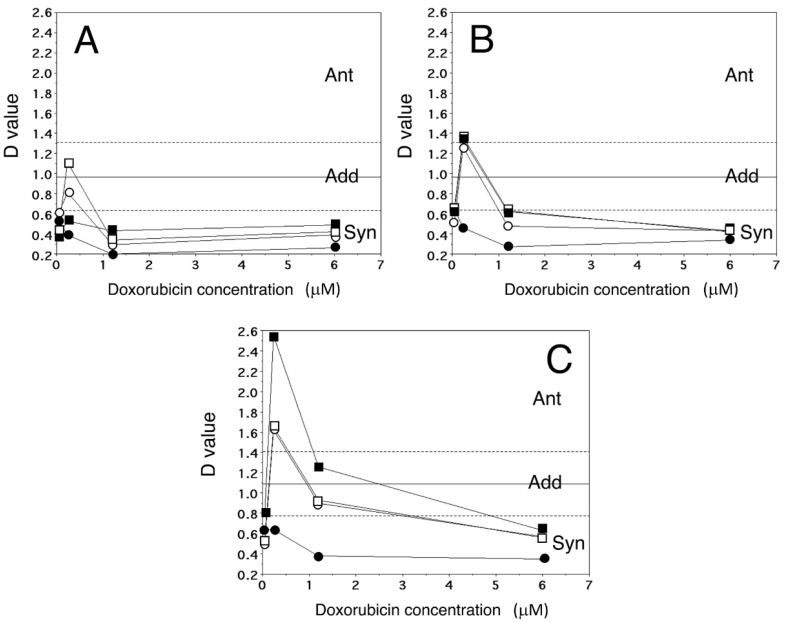
The graphs represent the D values (*n* = 4–7) obtained after combined treatment of A2780/DX3 cells with Dox and Lube S, R, or S/R (panels **A**, **B**, and **C**, respectively). The mean ± SD experimental D value for sham combinations was 1.10 ± 0.44. Ant, antagonism; Add, additivity; Syn, synergism. Lube concentrations: 0.005 µM (□), 0.05 µM (■), 0.5 µM (○), and 5 µM (●).

As reported in the previous section, A2780 and A2780/DX3 cells treated with Dox showed IC_50_s that were on average 0.018 ± 0.004 and 1.44 ± 0.53 µM, respectively, with a mean RI of 80. When A2780/DX3 cells were treated with combinations of different concentrations of Dox and Lube (both the enantiomeric forms and the racemate), we observed that Lube S synergized more than its enantiomer R and the racemic mixture (Figure 1). In no cases, synergism was observed treating cells with the higher Dox (30 µM) or Lube enantiomer (50 µM) concentrations, except for the combination of 30 µM Dox plus 5 µM Lube S/R (Tables S1–S3). In particular, 0.05 and 0.005 µM Lube S caused only 1.9% and 6.6% cell proliferation inhibition, respectively, in A2780/DX3 cells. At these concentrations, Lube S significantly synergizes with Dox as the anticancer drug IC_50_ was reduced by 56% and 59% (from 1.44 ± 0.53 µM without Lube S to 0.63 ± 0.29 µM and 0.59 ± 0.26 µM with 0.05 and 0.005 µM Lube S, respectively, *p* = 0.001), similar to the results obtained on A2780 cells [7].

Since our data demonstrate that, on the A2780/DX3 cells, Lube S displayed the highest effect compared with Lube R and Lube S/R, most of the further experiments described in this article have been performed with Lube S in combination with Dox.

### 2.3. Detection of Apoptosis

Three different drug combinations (1.2 µM Dox plus 0.5, 5, or 50 µM Lube S) were selected and used in cytofluorimetric tests to verify the effect of drug combinations on the apoptosis induction in A2780/DX3 cells. In all cases, D values correlated with those calculated for the antiproliferative activity (Table 3), suggesting a similar effect of drug combinations on these two processes.

It is also of note that a completely overlapping result was obtained with sensitive A2780 cells, using opportune drug concentrations: 0.2 µM Dox plus 0.5, 5, or 50 µM Lube S (Table S4).

### 2.4. Analysis of Dox Accumulation by Flow Cytometry

Lube enantiomers were also analyzed for their ability to counteract the efflux of Dox from A2780/DX3 cells to evaluate the MDR1 inhibition as a possible mechanism of action. The results of this activity in A2780/DX3 cells are summarized in Figure S2.

In our test conditions, only higher equitoxic concentrations (IC_50_ and IC_75_) of Lube S caused a significant increase in the ratio between Dox accumulation in sensitive A2780 and multi-drug-resistant A2780/DX3 of 6.5 (±1.7) and 6.7 (±0.7)-fold, respectively. Notably, in similar test conditions, the ratio between Dox accumulation in sensitive A2780 and multi-drug-resistant A2780/DX3 cells was 5.9 ± 1.0.

It is also important that Lube S did not cause accumulation of Dox in sensitive A2780, thus indirectly confirming the presence of the hypothesized mechanism of action involving the inhibition of MDR1 (Table 4).

### 2.5. Docking Studies

To investigate whether the mechanism underlying the synergism between Dox and Lube S affects the MDR1 activity, we performed a structure-based modeling study, taking advantage of the significant boost of insights gained in the latest years on MDR1 from Cryo-EM. To achieve this goal, dockings of both ligands were carried out using the ABCB1 structure complexed with taxol recently determined by Alam et al. [19]. In this structure, it is clear how a P-gp binder might fit into the ABCB1 internal cavity located between the two symmetrical halves composing the typical six-helix domains opened towards the cytoplasm. Moreover, if the binder is a substrate, it could be pulled out through an inward- to an outward-facing flip of the P-gp scaffold. Indeed, in previously published studies [20,21], we have already proposed that the “inward–outward facing” might be hampered by active inhibitors able to halt the same flipping.

Our synergistic hypothesis arises from the data showing that MDR1 can simultaneously host two binders, as demonstrated by the ABCB1 ability to bind elacridar, tariquidar, and zosuquidar with a 2:1 stoichiometric ratio [22,23]. Before any attempts to identify for Dox and Lube S any correct bindings to a P-gp site, we first ensured that both the crystallographic data and the applied docking protocol (see methods) were sound enough to reproduce the experimentally observed taxol pose in the Cryo-EM structure. Then the same ligand was submitted to redocking to ABCB1. Due to the extremely articulated taxol molecular scaffold, presenting highly diverse structural motifs (i.e., four fused and flexible rings, a high number of rotamers, chiral atoms, a cis amide bond, etc.), and the low resolution of the available three-dimensional data, similarity criteria based on a shaped and atom-based fitting were therefore used as scoring criteria to assess the quality of redockings. Indeed, the achieved taxol binding pose was very similar to the one experimentally determined, as proved by a TanimotoCombo coefficient equal to 0.837 (see Figures S3 and S4). Therefore, as long as this check was acquired, a two-step docking was then performed, in which Dox and Lube S were posed consequently.

**Figure 2:**
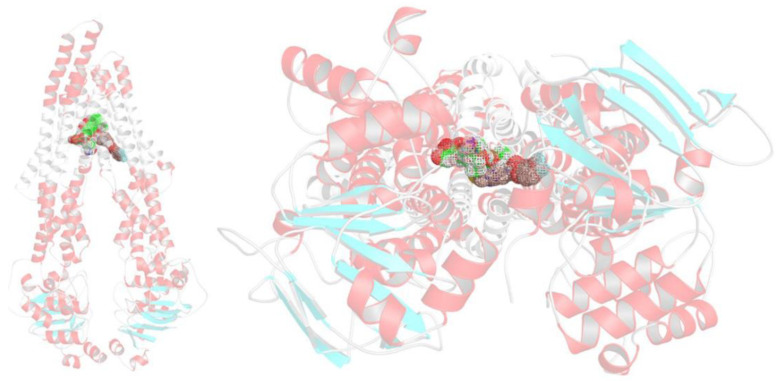
Binding mode of Dox (green carbon) and Lube S (brown carbon) to the MDR1. Front (**left**) and intracellular view (**right**) of the achieved poses. The transmembrane-spanning helices are depicted as white cartoons, ligands in meshes.

**Figure 3:**
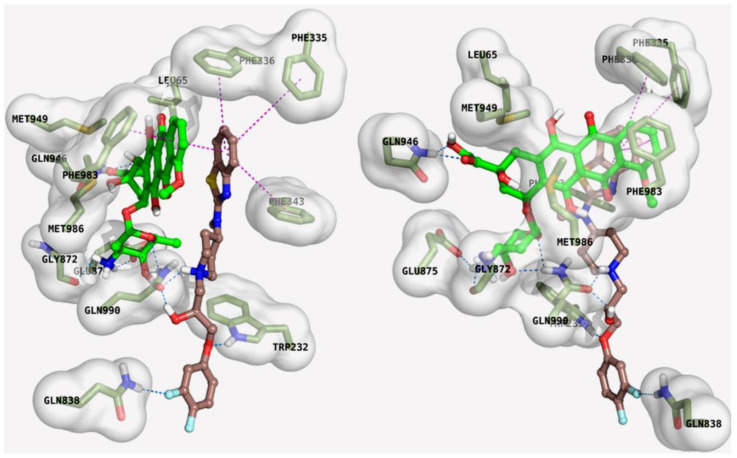
Front (**left**) and side (**right**) detailed view of Dox and Lube S docking. In the interaction pattern scheme, hydrogen and halogen bonds and π–π stackings are depicted in blue and magenta, respectively.

In this case, after the Dox binding in its specific site, Lube S occupied a secondary cleft, most likely of allosteric nature. Therefore, we decided to execute two-step dockings, where Dox was primarily posed and Lube S was after that proven to be able to occupy a secondary, most likely allosteric, cleft. This idea was essentially accomplished as it might be perceived from Figure 2 and Figure 3.

Indeed, both molecules might be easily accommodated into the “inward-facing” elbow helix binding site, with many ligand–protein and ligand–ligand interactions. This strongly supports the stability of the MDR1–Dox–Lube S complex that hampers the transmembrane domains flipping and, therefore, the “pulling out” of xenobiotics released with an “outward-facing” of the protein. In detail, Dox is anchored on the receptor surface, engaging a salt bridge with the charged nitrogen and Glu875, as well as with hydrogen bonds with Gln946 and Gln990. In contrast, the aromatic moiety embraces two-side face-to-face π–π stacking with Phe983 and Lube S. At the same time, different edge-to-face π–π stackings are gained by the benzothiazole ring system of the same Lube S with a cluster of aromatic residues, comprising Phe335, Phe336, Phe343; besides, several polar interactions with Gln838 and Gln990 are retrieved by the ligand-charged piperidine nitrogen, the hydroxyl group, and the fluorine atom (Figure 3).

As a figure of merit, favorable data are gained in the two consecutive docking steps (Table 5). Notably, regardless of the difference in FEB values, Dox and Lube S showed similar ligand efficacy in their binding to MDR1.

### 2.6. Influence of Lube S on MDR1 Expression

Since the accumulation of Dox in A2780/DX3 cells could also be due to a downregulation of MDR1 expression [24,25,26], the expression of this active transporter was evaluated after a 3-day exposure to different concentrations of Lube S.

**Figure 4:**
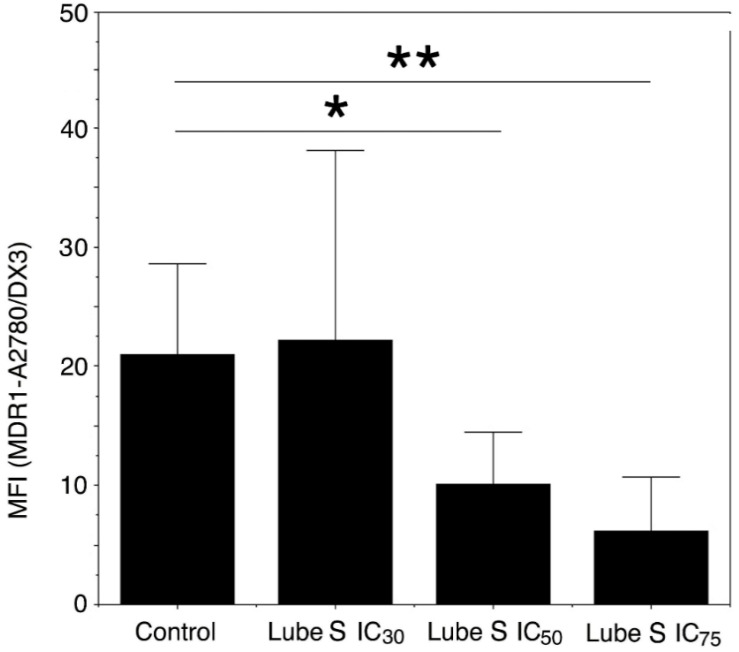
Expression of MDR1 after exposure to equitoxic concentrations of Lube S (IC_30_ = 3.35 µM; IC_50_ = 7.36 µM; IC_75_ = 23.6 µM). ANOVA for expression of MDR1, *p* = 0.0866. Lube S IC_50_ (*n* = 5) vs. control (*n* = 5), * *p* = 0.016; Lube S IC_75_ (*n* = 3) vs. control, ** *p* = 0.071, as evaluated by the Mann–Whitney test.

Our results indicate that the exposure to concentrations corresponding to IC_30_, IC_50_, and IC_75_ Lube S caused a trend toward a dose-dependent decrease in MDR1 expression on the cell membrane (ANOVA, *p* = 0.0866). When Lube S was applied at its IC_50_ and IC_75_, the decrease in MDR1 expression, as compared with untreated cells (Figure 4), became significant (IC_50_, *p* = 0.016) or showed a trend (IC_75_, *p* = 0.071).

### 2.7. Effect of Lube S and Dox on the Intracellular Oxidative Stress Damages in A2780/DX3 Cells

MDA is a stable product of lipid peroxidation; therefore, it can be used as an indirect measure of cumulative intracellular oxidative stress damage. MDA quantification was determined on A2780/DX3 cells treated with Lube S, Dox, or their combination.

Our data show that oxidative stress damages induced by 6 or 1.2 μM Dox are significantly improved in combination with 0.5 and 0.05 µM Lube S (Table 6). Conversely, 0.005 µM did not increment the intracellular oxidative stress damage. We verified a significant antagonistic or additive effect on the production of MDA (Table 6) instead.

### 2.8. Effect of Lube S on the Invasion of MDA-MB-231

Since the migration/invasion ability of A2780/DX3 was nearly absent, we studied the ability of Lube S alone to inhibit cell invasion in MDA-MB-231 cells using Matrigel containing Transwell chambers in the presence of its IC_30_, IC_50_, and IC_75_.

**Figure 5:**
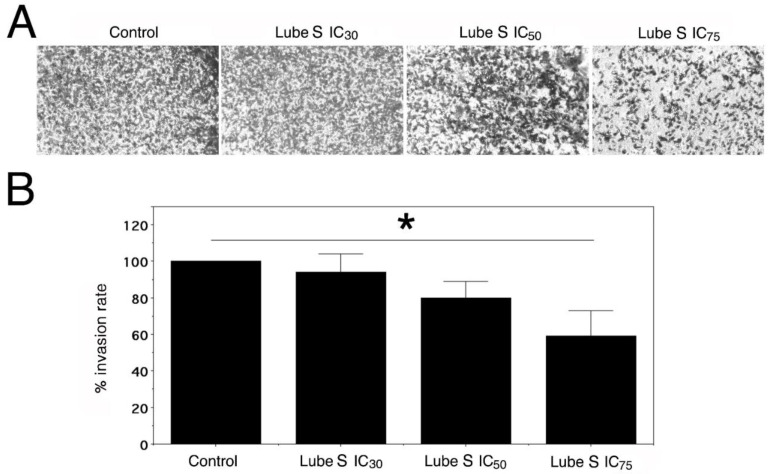
Panel (**A**) shows a representative image of invasive MDA-MB-231 cells at 40× magnification. Panel (**B**) shows the mean percentage ± SD (*n* = 3) vs. control of migrated cells after exposure to the equitoxic concentrations of Lube S (IC_30_ = 3.35 µM; IC_50_ = 7.36 µM; IC_75_ = 23.6 µM). ANOVA, *p* = 0.0053. IC_75_ vs. control, * *p* < 0.05 (Mann–Whitney test).

Our results show (Figure 5) that Lube S inhibited the motility of cells and the invasion process in a concentration-dependent manner (*p* = 0.0053) with about 41 ± 14% reduction in cell invasion at the higher concentration applied (*p* < 0.05).

### 2.9. NO Detection

The effect of drug combinations on intracellular NO synthesis was studied in A2780/DX3 cells. Single drugs Dox and Lube S caused a linear correlation between the applied concentrations and intracellular NO production. Moreover, while Lube S slightly inhibited the production of intracellular NO compared with untreated cells at all concentrations examined, only 30 µM Dox caused an increase in intracellular NO.

**Figure 6:**
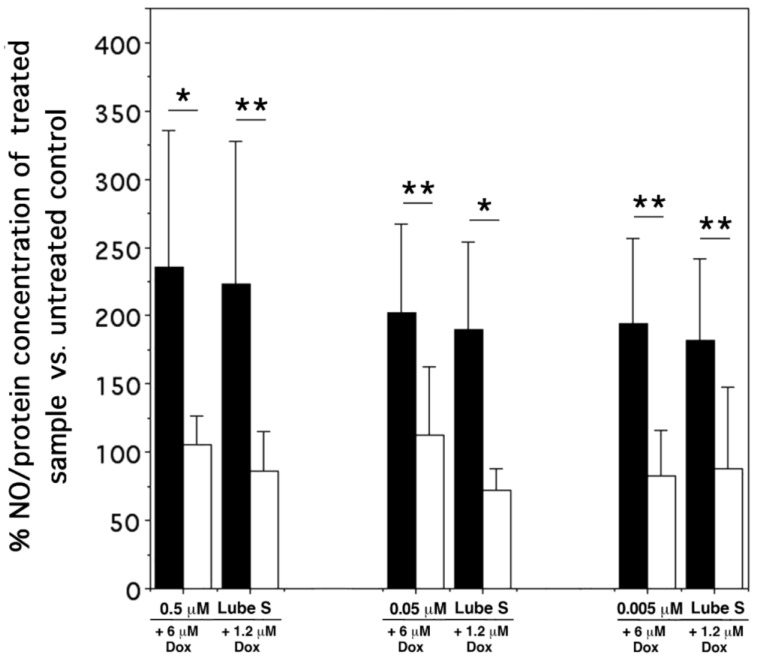
Bars represent the mean ± SD (*n* = 6) from the intracellular forecasted % NO concentration (⬛) vs. the observed % NO concentration (⬜) for each of the combined treatments applied to A2780/DX3 cells. The mean ± SD (*n* = 6) of absolute NO concentration was 0.845 ng NO/mg protein, while the mean linear correlations for single drug administration of Dox and Lube S were represented by the equations: y = 2.0761x + 81.443 (r^2^ = 0.999, *p* = 0.0007) and y = 40.601x + 70.289 (r^2^ = 0.999, *p* = 0.009). The comparison between groups of data was performed by the Mann–Whitney test. * *p* < 0.01; ** *p* < 0.05.

In all tests, when Dox and Lube S were combined, the observed concentration of NO, primarily expressed as ng NO/mg protein and then as a percentage of the untreated control (Figure 6), was significantly lower than that predicted based on NO production obtained by single Dox and Lube S treatment.

## 3. Discussion

In a previous paper [7], we demonstrated that the combination of Lube S with Dox or paclitaxel displayed a remarkable synergism at sub-micromolar concentrations, with a more marked effect on A2780 cells. These results suggested that Lube S could potentiate in vivo Dox anticancer activity at very lower concentrations than those inducing direct cytotoxicity.

In the present study, we expanded our study to A2780/DX3 cells, which display multidrug resistance linked to the overexpression of the MDR1 transporter [16,17]. As previously observed in sensitive cells, 0.005 µM Lube S significantly synergizes with Dox on A2780/DX3 cells, reducing anticancer drug IC_50_ by about 59%, starting from a Dox alone IC_50_ of 1.44 ± 0.53 µM. At the applied concentration, Lube S alone showed inhibition of cell proliferation lower than 2%.

Moreover, in the past paper, we observed that 0.005 µM Lube S was about 50 times lower than human plasma concentration related to QTc interval prolongation (>100 ng/mL), suggesting a possible repositioning of Lube S as an adjuvant for Dox or other anticancer drugs. On the other hand, the cell proliferation inhibition obtained in the present study, together with the apoptosis triggering, strengthens our previous observations, allowing us to hypothesize a possible use of Lube S as adjuvant, also in the case of cells with acquired multidrug resistance.

As we have already demonstrated, Lube S antagonizes CaM activities, and this mechanism may contribute to its observed chemosensitizing properties [6]. CaM is a ubiquitous protein that modulates the activity of several proteins, acting as a Ca^2+^-sensor [27]. Several studies have demonstrated that some anticancer drugs may bind to CaM, while some CaM ligands may act as chemosensitizing agents [28,29,30]. More importantly, loperamide and trifluoperazine, two CaM ligands, share similar structural features with Lube S. Interestingly, Lube S displays antidiarrheal and adjuvant antibacterial activities like those of loperamide [31].

Based on our previous results and literature information [6,27,28,29,30], we speculate that the inhibition of CaM may play a fundamental role in determining the synergistic effects of the combination of Lube S and Dox not only on sensitive A2780 cells but also on multi-drug-resistant A2780/DX3 cells. On the other hand, our resistant cells exhibit a mechanism of resistance that could be involved in the potentiating action of Lube S. A2780/DX3 cells are characterized by an overexpression of MDR1 [17] that implies a rapid efflux of Dox and other unrelated anticancer drugs. Our results show that Lube S may increase Dox accumulation in the cells by inhibiting MDR1 activity. Moderate stereoselectivity was found, with Lube S being significantly more potent than its enantiomer (Lube R) and the corresponding racemate (Lube S/R). This activity is nearly immediate and probably depends on the direct binding of Lube S to MDR1. Docking simulation studies on the ABCB1 Cryo-EM structure supported the hypothesis that Lube S forms a stable MDR1–Dox–Lube S complex, which hampers the protein transmembrane domain flipping and blocks the efflux of Dox from resistant A2780/DX3 cells.

Furthermore, Lube S also slightly reduces in a dose-dependent manner (we observed only a trend) the MDR1 expression, thus contributing to drug accumulation. Although not always statistically significant, this activity was however expressed after a long exposure interval. It is known that different drugs may downregulate MDR1 activity [24,25,26], including verapamil, which acts through a transcriptional mechanism [26]. Even if the mechanism of MDR1 downregulation exerted by Lube S remains unclear, its double-level action on MDR1 transport activity suggests a new strategy for counteracting drug resistance linked to the MDR1-dependent multidrug resistance mechanism.

On the other hand, our data also indicate that these mechanisms are significant only at the higher allegedly toxic concentrations used [7]. This fact should be considered when performing in vivo experiments with suitable cancer-targeted Lube S formulations.

The same considerations hold for the invasion inhibition exerted by Lube S. In fact, also in this case, a clear and significant effect on cell migration/invasion was observed at the higher concentration used. Nevertheless, our data were obtained in a different cell line (MDA-MB-231) because A2780/DX3 cells do not display invasiveness, thus resulting only in a further mechanism for Lube S activity.

We also analyzed the activity of Lube S co-administered with Dox as an enhancer of oxidative stress damage. Our results show that the Dox action mechanism could be differently modified based on Lube S concentrations. At the higher Lube S (allegedly toxic) concentrations, we observed a significant synergism in the oxidative stress damage triggered by Dox. Conversely, at the lowest Lube S concentration (0.005 µM), we observed an additive or antagonistic interaction at a dose of 1.2 or 6 µM Dox, respectively. This result suggests using a low dose of Lube S as a potentiating agent for Dox treatment, considering that Dox cardiac toxicity is partly due to oxidative stress on cardiomyocytes [32,33]. In other words, at least at its lower concentration, Lube S seems not to potentiate the oxidative stress damages, preserving its action on MDR1 and the other phenomena analyzed here.

Finally, based on the past demonstration that Lube S can reduce glutamate-activated NO synthesis [2], we evaluated the pharmacological activity of Dox and Lube S on the intracellular levels of this molecule.

NO plays multiple and sometimes controversial actions in cancer biology, particularly ovarian cancer [34]. While it is known that at low concentrations NO promotes tumor growth [35], at higher (exogenous) concentrations, it becomes tumoricidal [36] and can sensitize tumor cells to chemotherapy [37,38]. It has been demonstrated that a high expression of iNOS (inducible NO synthase, one of the isoforms of NOS) correlates with a worse prognosis in ovarian carcinoma [39,40], being associated with more invasive ovarian cancers. On the other hand, not only iNOS levels but oxygen availability, necessary for NO synthesis, is also relevant for generating this mediator, thus implying that the level of iNOS does not always correlate with the level of NO.

Furthermore, it has been demonstrated that omental adipose stroma cells induce in ovarian cancer cells NO synthesis with a consequent augmented proliferation and resistance to paclitaxel [41]. Other studies suggest that low basal NO concentrations in ovarian cancer cells are requested for ovarian cancer survival [42], while Fraser et al. [43] showed that inhibition of sGC (an enzyme involved in the NO/sGC/cGMP-dependent pathways) increases apoptosis through an increase in the p53 protein.

Overall, these observations make the role of NO quite controversial but also suggest that it could be better defined by considering its origin (intra- or extracellular), the type of tumor considered, the combined effect of the other NO signaling actors, and the tumor microenvironment.

We do not know whether the selection in vitro procedures used to obtain A2780/DX3 cells may have also altered their genetic makeup linked to NO induction, synthesis, and regulation. Thus, based on the information mentioned before, we hypothesize that a further decrease in NO intracellular content (in case of combined treatment) may correlate with increased anticancer activity of Dox, at least in our cellular model.

Our results, which demonstrate a true synergism of Lube S and Dox in terms of inhibition of cell proliferation and apoptosis, support this hypothesis, thus providing (at best) another possible mechanism of action for the potentiating activity of Lube S in A2780/DX3 multidrug-resistant cells treated with Dox. Nevertheless, the regulation of NO content by Lube S and the contribution of the anti-ischemic drug to Dox resistance (multidrug resistance) remain unclear, and more in-depth research is needed to clarify this point.

In conclusion, Lube S may synergize in vitro with Dox through several mechanisms. Although some mechanisms can be activated only at relatively high concentrations, possibly yielding toxic effects, those activated at low concentrations could be exploited for improving Dox action on cancer and also in resistant cells possibly selected by chemotherapy.

Finally, the observation that in human normal cells the antiproliferative activity of Lube S was much lower than that observed in tumor cells further suggests a possible use of this anti-ischemic drug for the potentiation of anticancer activity of Dox.

## 4. Materials and Methods

### 4.1. Cells

Human lung adenocarcinoma A549 and ovarian carcinoma A2780 cells sensitive to Dox were maintained in culture in RPMI 1640 medium in the presence of 10% fetal calf serum (FCS), 1% glutamine, and 1% penicillin–streptomycin (complete medium). Human breast cancer MDA-MB-231 and hepatocarcinoma HepG2 were maintained in DMEM complete medium. Furthermore, multi-drug-resistant A2780/DX3 cells (provided by Dr. Y. M. Rustum and obtained by exposure to increasing concentrations of Dox) were maintained in RPMI 1640 complete medium in the presence of 0.1 µM Dox. Three–four days before performing the assays, Dox was removed from the medium.

### 4.2. Chemicals

Lube S, its enantiomer (Lube R), and their racemate (Lube S/R) [44] were stocked at 100 mM in DMSO and frozen at −20 °C. When used, they were reconstituted at 1 mM in distilled water and further diluted in distilled water containing 2% DMSO (final concentrations: 0.005–100 µM). Dox (Adriblastina, Pfizer Italia Srl, Latina, Italy) was prepared starting from its clinical formulation and dissolved in normal saline (final concentrations: 0.048–30 µM).

### 4.3. In Vitro Studies of Inhibition of Cell Proliferation

Human ovarian adenocarcinoma multi-drug-resistant A2780/DX3 cells and their sensitive counterpart A2780 were plated at 4000 and 2700/well, respectively, into 96-well microtiter plates for 8 h. Similarly, MDA-MB-231, HepG2, and A549 cells were plated at 2900, 5500, and 1250/well, respectively. As normal noncancer cell targets, we used lymphocytes obtained from the peripheral blood (PBL) of three healthy volunteers isolated by gradient centrifugation (Lympholyte-H Cell Separation Media, EuroClone, Pero, MI, Italy) and stimulated with 1% phytohemagglutinin and 100 U/mL recombinant IL-2. One week after, stimulation cells were ready for use at 15,000/well.

Tumor and nontumor cells were then treated with Lube S, R, or S/R (PBL only with Lube S) to calculate their IC_5_, IC_10_, IC_30_, IC_50_, and IC_75_. They were administered in duplicate for a minimum of five concentrations (4- or 10-fold serial dilutions, maximal volume/well: 200 μL). Three days later, 50 μL of 3-(4,5-dimethylthiazol-2-yl)-2,5-diphenyltetrazolium bromide (MTT, Sigma, St. Louis, MO, USA) solution (2 mg/mL in PBS) was added, and plates were treated as already described [45].

The effect of combinations of Lube S, R, or S/R, and Dox was studied in multi-drug-resistant A2780/DX3 cells, after continuous exposure to a single drug or drug combination for 72 h. Cells were plated in 96-well flat-bottomed microtiter plates at 4200/well and centrifuged at 1100 rpm for 2 min, and single or combined drugs were added after 8 h. Drugs, at opportune 20X concentrations, were mixed at 1:1 just before the addition to the wells, containing a final volume of 200 μL. The MTT assay was performed after 3 days as described before.

### 4.4. Detection of Apoptosis by 4′,6-Diamidino-2-Phenylindole (DAPI) Test

A2780/DX3 cells were plated, as described in the previous section, in 24-well plates for about 6–8 h. Cells were then treated with selected combinations of Lube S and Dox mixed (1.2 µM Dox plus 50, 5, and 0.5 µM Lube S). After 72 h, floating and adherent cells were harvested and washed twice with cold phosphate-buffered saline (PBS). Pellets were then fixed with 100 μL of 70% ethanol in PBS and maintained at 4 °C. To determine the percentage of cells displaying segmented nuclei (apoptotic cells), cells were first stained with 6 μL of the DNA specific dye DAPI in water (10 μg/mL) and then observed and counted (at least 200) by epifluorescence microscopy.

### 4.5. Cytofluorimetric Study of Intracellular Accumulation of Dox

A2780 and A2780/DX3 cells were plated in 25 cm^2^ flasks at 1 × 10^6^ and 1.5 × 10^6^ cells/flask, respectively, in 10 mL. After 24 h, once a confluence of about 75–85% was reached, cells were pretreated with Lube (S, R, or S/R) at their IC_10_, IC_30_, IC_50_, and IC_75_, as calculated in the MTT assay. After 2 h, Dox was added in a small volume to reach the final concentrations of 10 µM in A2780 and A2780/DX3 cells, and the incubation was performed for an additional 2 h. Cells were then harvested after treatment with trypsin at 37 °C for 5 min, washed twice with cold PBS, and fixed for 20 min with 3.7% paraformaldehyde in PBS containing 2% sucrose. Once washed twice with PBS containing 2% FCS, cells were pelleted and concentrated in the same medium. Untreated cells and control cells treated with Lube S (negative controls) or Dox (positive control) were also made.

The intracellular fluorescence intensity was determined by flow cytometry (FACScan, BD Biosciences, Milano, Italy) using 488 nm excitation and 575 nm bandpass filter for Dox detection. Values represent the ratio between the mean fluorescence intensity (MFI) of cells treated with Dox and Lube and cells treated with only Dox, here considered as reference (Dox value = 1).

### 4.6. Study of MDR1 Expression

A2780/DX3 cells were treated for 72 h with Lube (S, R, S/R) at their IC_30_, IC_50_, and IC_75_ to study their possible downregulating activity of MDR1 expression. Once harvested and washed three times with PBS plus 2% FCS, cells were pelleted at 2.5 × 10^5^ and incubated at 4 °C with 25 μL of anti-MDR1 MM4.17 [46] monoclonal antibody. After washing cells three times with PBS plus 2% FCS, they were incubated again with an FITC-labelled goat anti-mouse IgG (Jackson ImmunoResearch Laboratories, West Grove, PA, USA). After three more washes in washing buffer, alive cells (gated) were analyzed by flow cytometry (FACScan, BD Biosciences, Milano, Italy).

### 4.7. Cell Invasion Assay

The effect of Lube S on the invasion process of MDA-MB-231 cells was evaluated in vitro using the Transwell chambers (Corning Costar, Cambridge, MA, USA). Matrigel (Corning, Bedford, MA, USA), diluted at 0.3 mg/mL in DMEM medium without FCS, was applied onto the 8 mm pore size polycarbonate membrane filters of the Transwell chambers, and incubated at 37 °C for 16 h, allowing its polymerization. MDA-MB-231 cells, at 4 × 10^4^ cells/well and suspended in DMEM medium without FCS, were then seeded on the upper part of the chamber at a final volume of 200 μL. The bottom chamber was filled with 800 μL of DMEM medium containing 10% FCS. After 2 h, cells were treated with Lube S at its IC_30_, IC_50_, and IC_75_ for 16 h.

Cells migrating to the lower chamber through the Matrigel basement were fixed with paraformaldehyde 3.7%, stained with 0.1% crystal violet, and photographed. Invasion of MDA-MB-231 cells was defined in terms of percentage of invasive cells vs. untreated controls after counting three randomly selected fields.

### 4.8. Evaluation of Intracellular Malondialdehyde Level as a Marker of Oxidative Stress Damage

A2780/DX3 cells were treated with Dox (0.24, 1.2, 6, 30 µM) and Lube S (0.005, 0.05, and 0.5 µM) alone or combined, as described for the MTT assay. After 1 h, cells were harvested, washed twice with cold PBS, counted, pelleted, and frozen at −20 °C.

The concentration of malondialdehyde (MDA), a breakdown product of lipid peroxides, was evaluated by the thiobarbituric acid reactive substance (TBARS) assay. This test is based on the reaction of MDA with thiobarbituric acid (TBA). The TBARS solution contained 15% trichloroacetic acid in 0.25 M HCl and 26 mM TBA acid. Briefly, cells were homogenized in 300 µL of Milli-Q water and sonicated twice for 10 s at 4 °C. Afterwards, 600 µL of TBARS solution was added to the sample, and the mix was incubated for 40 min at 95 °C. The sample was centrifuged at 14,000 rpm for 2 min, and the supernatant was analyzed spectrophotometrically at 532 nm. The MDA concentration was calculated by a calibration curve obtained using different MDA concentrations between 0 and 2 µM. Data were normalized on the total protein content, evaluated by the Bradford method, using BSA as the standard protein [47,48].

### 4.9. Molecular Modelling Methods

The Lube S X-ray structure was used for dockings [49], while Dox three-dimensional was retrieved from PubChem [50]. The proper ionization was then assigned with the *fixpka* complement of QUACPAC (QUACPAC 2.1.0.4: OpenEye Scientific Software, Santa Fe, NM), and thereafter, 10,000 steps of steepest descent minimization using the universal force field was then performed with Open Babel [51]. The MDR1 Cryo-EM structure (chain A from the pdb code 6QEX) was prepared with the Protein Preparation Wizard interface of Maestro [52], removing the ligand and taxol molecules, adding hydrogen atoms, optimizing their position, and assigning the ionization states of acid and basic residues according to PROPKA prediction at pH 7.0. Electrostatic charges for protein atoms were loaded according to the AMBER UNITED force field [53], while the *molcharge* set of QUACPAC was used to achieve Marsili-Gasteiger charges for the inhibitors.

Taxol was redocked using affinity maps calculated on the apo state of the protein on a 0.375 Å spaced 100 × 100 × 100 Å^3^ cubic box, having the barycenter on the same taxol fragment, and comparison with the experimental binding pose was scored using an ROCS shape matching algorithm (ROCS 3.4.0.4: OpenEye Scientific Software, Santa Fe, NM). A solvent was explicitly considered using the proper parametrization of water contribution according to the relative AUTODOCK hydration force field [54], and the population size and the number of energy evaluation figures were set to 300 and 10,000,000, respectively. Docking of Dox was then performed throughout 1000 runs of the Lamarckian genetic algorithm (LGA) implemented in AUTODOCK 4.2.6 [55] using the GPU-OpenCL algorithm version [56], and the best free energy pose as scored by AUTODOCK was selected to create the Doxo–MDR1 complex, and once this instance was achieved, novel maps were again calculated to perform Lube S docking.

### 4.10. Detection of NO Intracellular Levels in A2780/DX3 Cells

A2780/DX3 cells were plated (2.33 ×10^4^/mL) into 180 cm^2^ flasks suspended in 60 mL of RPMI 1640 medium. After 8 h, cells were treated with single drugs Dox (0.24, 1.2, 6, and 30 µM) and Lube S (0.005, 0.05, and 0.5 µM) or their combinations (6 and 1.2 µM Dox plus 0.005, 0.05, and 0.5 µM Lube S). After 18, h cells were treated with trypsin, washed with PBS, and resuspended in 150 μL PBS. Proteins were extracted by sonication and dosed by the Bradford assay [48].

NO production was assayed by the Griess reagent using the Nitric Oxide (total) detection kit (Enzo Life Science, Lausen, Switzerland). The absorbance was measured at 545 nm on a microplate reader. NO concentration was determined using a dilution of a nitrate standard, while the final NO concentration was related to the concentration of intracellular proteins.

### 4.11. Data Analysis and Statistics

The resistance index (RI) was calculated for each drug as the ratio between IC_50_ of resistant A2780/DX3 cells and the corresponding wild-type A2780 cells.

The isobole method [57] was applied to analyze the effects of combination treatment. In particular: for drug combinations of A and B, the combination index D was calculated by the equation:

D = Ac/Ae + Bc/Be(1)

where Ae and Be were the concentrations of compounds A and B that gave alone the same observed magnitude of effect, while Ac and Bc were the compound concentrations used in the examined combination [58]. When (a) D = 1, the effect of the combination is considered additive; (b) D < 1, the effect is considered synergistic; and finally, (c) D > 1, the effect of the combination is considered antagonistic.

The experiments were repeated four to eight times. The experimental D value for additivity (sham combination) was calculated using combinations of serial dilutions of the single drugs utilized for the experiments.

The Mann–Whitney and ANOVA tests were used for the statistical analysis of data.

## Figures and Tables

**Table 1:** Antiproliferative activity (IC_50_, μM) of Dox, Lube S, Lube R, and Lube S/R in cell lines with different histologic origins.

Drug/Compound	Cell Lines				
	A2780	A2780/DX3	HepG2	MDAMB231	A549
Dox	0.018 ± 0.004 ^a^	1.4 ± 0.5	0.127 ± 0.018	0.053 ± 0.020	0.058 ± 0.014
Lube S	4.7 ± 1.5	6.5 ± 2.9	7.4 ± 1.3	7.4 ± 2.0	28.8 ± 10.0
Lube R	12.0 ± 2.3	18.6 ± 0.8	6.1 ± 1.3	16.2 ± 2.1	40.5 ± 10.1
Lube S/R	8.6 ± 2.0	29.6 ± 4.0	6.3 ± 1.9	8.6 ± 1.5	29.8 ± 9.2

^a^ IC_50_s (μM) are expressed as mean ± SD of 4–8 data.

**Table 2:** Equitoxic concentrations (μM) of Lube S, Lube R, and Lube S/R in A2780/DX3 resistant cells.

Drug/Compound	A2780/DX3				
	IC_5_ ^a^	IC_10_	IC_30_	IC_50_	IC_75_
Lube S	0.29	0.42	1.7	6.5	30.8
Lube R	0.65	1.5	7.3	18.6	39.2
Lube S/R	0.10	0.50	12.5	29.6	58.5

^a^ IC values were calculated using the mean curves obtained for the treatment of A2780/DX3 cells with stated compounds and expressed as μM concentrations.

**Table 3:** Correlation between D values for the antiproliferative activity and apoptosis ^a^.

LubeS (μM)	D Value	
	MTT	Apoptosis
	Doxorubicin (1.2 μM)	Doxorubicin (1.2 μM)
50	0.94 ± 0.17	1.02 ± 0.01
5	0.20 ± 0.06	0.69 ± 0.26 ^b^
0.5	0.29 ± 0.11	0.39 ± 0.17 ^c^

^a^ Apoptosis was detected by DAPI staining and counting of cells presenting segmented nuclei (*n* = 5–6, as an example of representative images of fragmented nuclei; see [18]). ^b^ *p* = 0.0455 vs. 1.00 ± 0.33 (D value for sham combination, Mann–Whitney test). ^c^ *p* = 0.0019 vs. 1.00 ± 0.33 (Mann–Whitney test).

**Table 4:** Percent modification of Dox accumulation in A2780 cells after coadministration of equitoxic concentration of Lube S.

Dox + IC_10_ Lube S	Dox + IC_30_ Lube S	Dox + IC_50_ Lube S	Dox + IC_75_ Lube S
−12 ± 3% ^a^	−4 ± 9%	−11 ± 8%	−13 ± 12%

^a^ Data (mean ± SD) express the percent modification of Dox accumulation (*n* = 3–4).

**Table 5:** Summary of the docking results.

Drug	FEB ^a^	ΔE ^b^	EFF ^c^	POP ^d^
Dox	−13.15	0.00	−0.337	127/1000
Lube S	−10.48	0.00	−0.350	73/1000

^a^ FEB, free energy of binding. ^b^ ΔE, energy difference between the selected pose and the relative global minimum. ^c^ EFF, ligand efficacy. ^d^ POP, cluster member population.

**Table 6:** D values for MDA production after treatment with Dox and Lube S combination.

Dox Concentration (μM)	Lube S Concentration (μM)		
	0.5	0.05	0.005
6	0.26 ± 0.13	0.14 ± 0.02	1.7 ± 0.7
	*p* < 0.001	*p* < 0.001	*p* = 0.004
	Syn	Syn	Ant
1.2	0.40 ± 0.26	0.4 ± 0.4	0.7 ± 0.4
	*p* = 0.001	*p* = 0.02	*p* = 0.036
	Syn	Syn	Add

Note: The mean ± SD experimental D value for the sham combination was: 0.80 ± 0.12 (*n* = 8). Ant, antagonism; Add, additivity; Syn, synergism.

## Data Availability

Not applicable.

## Supplementary Materials

The following supporting information can be downloaded at: https://www.mdpi.com/article/10.3390/molecules27227870/s1, Figure S1: Structures of lubeluzole (Lube S: R^1^ = OH, R^2^ = H) and its enantiomer (Lube R: R^1^ = H, R^2^ = OH); Figure S2: Bars represent the mean ± SD (n = 5–6) of the ratios between the MFI of Doxo plus Lube S, R, or S/R and the MFI of Doxo alone (Relative Dox MFI) in A2780/DX3 cells; Figure S3: Overlay of the cryoEM (pink carbons) and redocked (yellow carbons) taxol binding modes. The transmembrane spanning helices are depicted as white cartoons, ligands as balls and sticks; Figure S4: Sideview of the cryoEM (pink surface) and redocked (yellow surface) taxol binding modes. Table S1. D values for the combined experiments with doxorubicin and Lube S in A2780/DX3 cells. Table S2. D values for the combined experiments with doxorubicin and Lube R in A2780/DX3 cells. Table S3. D values for the combined experiments with doxorubicin and Lube S/R in A2780/DX3 cells. Table S4. Correlation between D values for the antiproliferative activity and apoptosis in sensitive A2780 cells.
