# Supplementary material for: Lubeluzole Repositioning as Chemosensitizing Agent on Multidrug-Resistant Human Ovarian A2780/DX3 Cancer Cells
Source: Molecules. 2022 Nov 15;27(22):7870. doi: 10.3390/molecules27227870 (PMC9695310; doi:10.3390/molecules27227870)
Supplement: Supplementary file 1 [file molecules-27-07870-s001.zip › molecules-1921846-supplementary.pdf]

Supplementary Materials

Lubeluzole Repositioning as Chemosensitizing Agent on Multidrug-Resistant Human Ovarian A2780/DX3 Cancer Cells

Maurizio Viale [1,*], Giovanni Lentini [2], Rosaria Gangemi [1], Patrizio Castagnola [1], Gualtiero Milani [2], Silvia Ravera [3], Nadia Bertola [3], Antonio Carrieri [2] and Maria Maddalena Cavalluzzi [2]

[1] UOC Bioterapie, IRCCS Ospedale Policlinico San Martino, Largo R. Benzi 10, 16132 Genova, Italy
[2] Dipartimento di Farmacia-Scienze del Farmaco, Università degli Studi di Bari 'Aldo Moro', Via E. Orabona 4, 70126 Bari, Italy
[3] Dipartimento di Medicina Sperimentale, Scuola di Scienze Mediche e Farmaceutiche, Università di Genova, Via De Toni 14, 16132 Genova, Italy
* Correspondence: maurizio.viale@hsanmartino.it; Tel.: +39-01-0555-8320

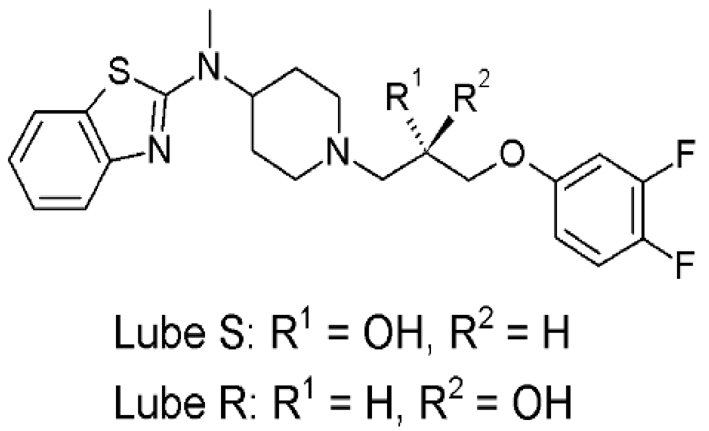

Figure S1. Structures of lubeluzole (Lube S: R^1 = OH, R^2 = H) and its enantiomer (Lube R: R^1 = H, R^2 = OH).

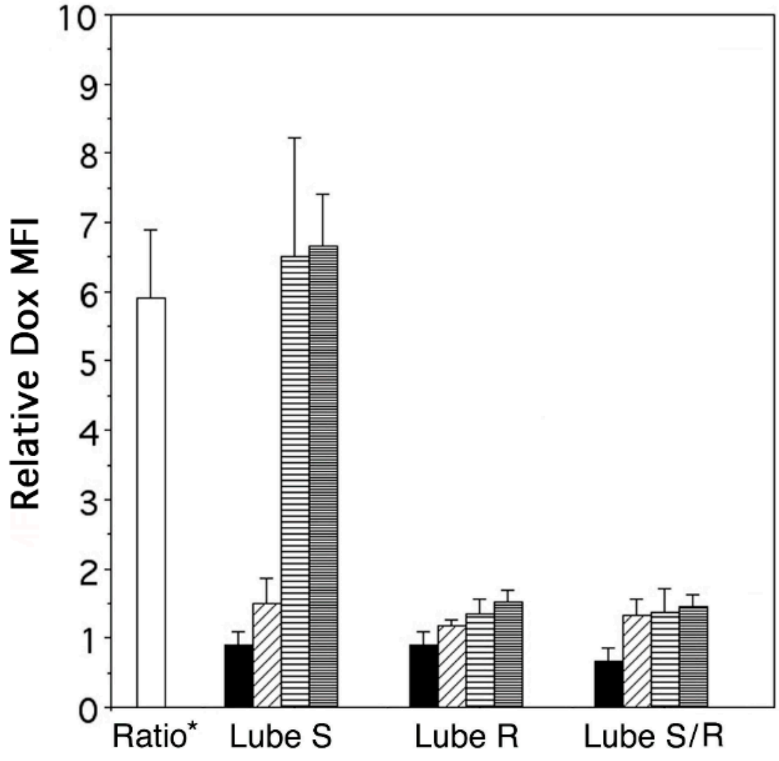

Figure S2. Bars represent the mean ± SD (n = 5 - 6) of the ratios between the MFI of Doxo plus Lube S, R, or S/R and the MFI of Doxo alone (Relative Dox MFI) in A2780/DX3 cells. Equitoxic Lube applied concentrations: IC_{10} (■); IC_{30} (▨); IC_{50} (▤); IC_{75} (▤); *Ratio A2780/(A2780/DX3) for Dox (□).

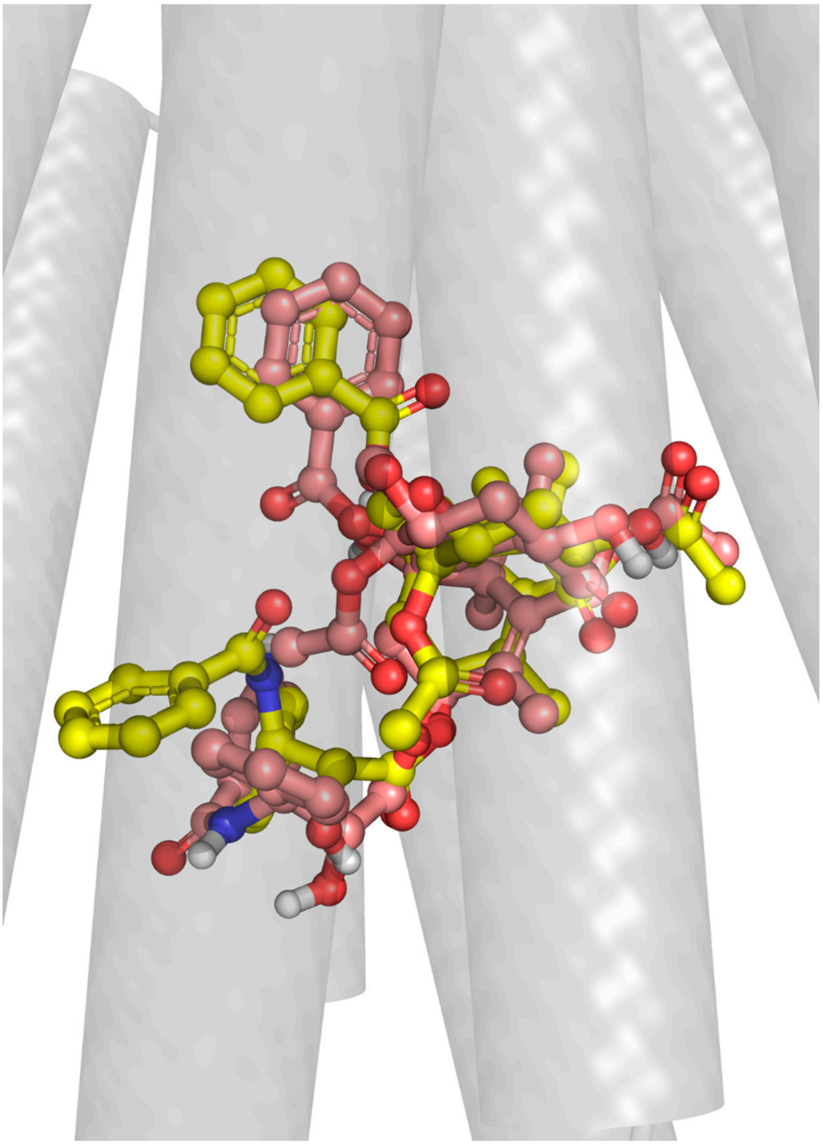

Figure S3. Overlay of the cryoEM (pink carbons) and redocked (yellow carbons) taxol binding modes. The transmembrane spanning helices are depicted as white cartoons, ligands as balls and sticks

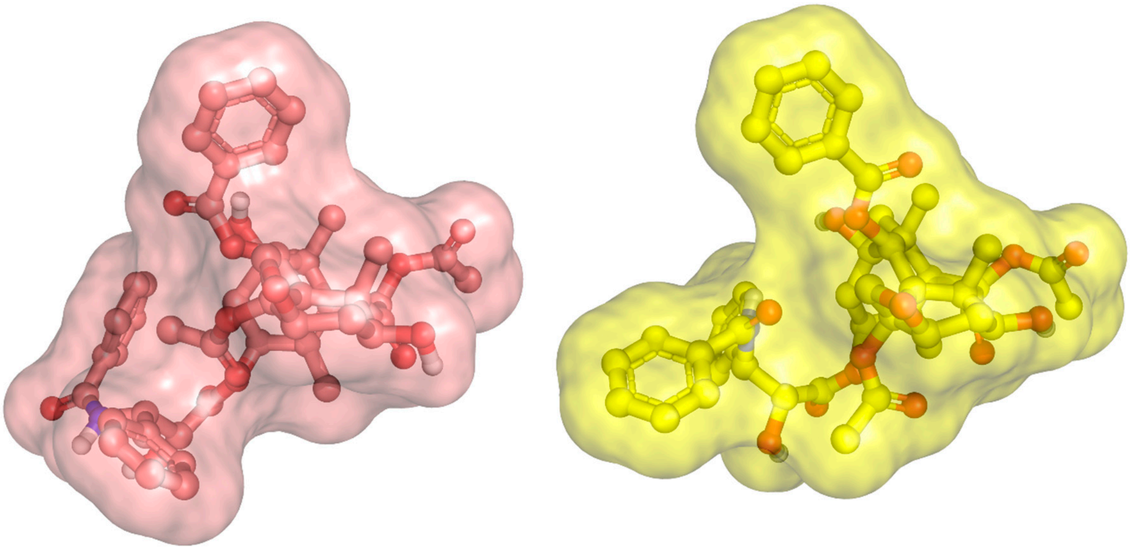

Figure S4. Sideview of the cryoEM (pink surface) and redocked (yellow surface) taxol binding modes.

Table S1. D values for the combined experiments with doxorubicin and Lube S in A2780/DX3 cells.

**Lube S**	**Doxo**				
	**30**	**6**	**1.2**	**0.24**	**0.048**
**50**	1.74±0.34[a] Ant	1.02±0.17 Add	0.94±0.17 Add	0.93±0.18 Add	0.91±0.19 Add
**5**	1.14±0.14 Add				
**0.5**	0.77±0.27 Add				
**0.05**	0.82±0.28 Add				
**0.005**	0.92±0.31 Add				

[a] The experimental D value for the sham combination was 1.10 ± 0.44.
Ant, antagonism; Add, additivity.

Table S2. D values for the combined experiments with doxorubicin and Lube R in A2780/DX3 cells.

Lube R	Doxo				
	**30**	**6**	**1.2**	**0.24**	**0.048**
**50**	1.74±0.13[a] Ant	1.12±0.01 Add	1.02±0.01 Add	1.00±0.01 Add	1.00±0.01 Add
**5**	0.81±0.31 Add				
**0.5**	1.11±0.12 Add				
**0.05**	1.24±0.09 Add				
**0.005**	1.17±0.14 Add				

[a] The experimental D value for the sham combination was 1.10 ± 0.44.
Ant, antagonism; Add, additivity.

Table S3. D values for the combined experiments with doxorubicin and Lube S/R in A2780/DX3 cells.

Lube S/R	Doxo				
	30	6	1.2	0.24	0.048
**50**	1.31±0.19[a] Ant	0.95±0.06 Add	0.90±0.06 Add	0.89±0.06 Add	0.91±0.05 Add
**5**	0.51±0.22 Syn				
**0.5**	1.14±0.09 Add				
**0.05**	1.25±0.45 Add				
**0.005**	1.08±0.40 Add				

[a] The experimental D value for the sham combination was 1.10 ± 0.44.
Ant, antagonism; Add, additivity; Syn, synergism.

Table S4. Correlation between D values for the antiproliferative activity and apoptosis in sensitive A2780 cells[a].

	D value	
	MTT	Apoptosis
	Doxorubicin 1.2 μM	Doxorubicin 1.2 μM
**50**	1.08±0.27<br>Add<br>NS	1.22±0.06<br>Add<br>NS
**5**	0.38±0.10<br>Syn<br>p<0.001	0.74±0.21<br>Syn<br>p<0.001
**0.5**	0.30±0.08<br>Syn<br>p<0.001	0.78±0.24<br>Syn<br>p<0.001

[a] Apoptosis was detected by DAPI staining and counting of cells presenting segmented nuclei. The D value for MTT assay was 1.09±0.34 while the D value for apoptosis was 1.11±0.32. The Mann–Whitney test was used for statistical analysis.

Add, additivity; Syn, synergism.
